# Efficient Inkjet Printing of Graphene-Based Elements: Influence of Dispersing Agent on Ink Viscosity

**DOI:** 10.3390/nano8080602

**Published:** 2018-08-08

**Authors:** Lucja Dybowska-Sarapuk, Konrad Kielbasinski, Aneta Arazna, Konrad Futera, Andrzej Skalski, Daniel Janczak, Marcin Sloma, Malgorzata Jakubowska

**Affiliations:** 1 Faculty of Mechatronics, Warsaw University of Technology, Andrzeja Boboli 8, 02-525 Warsaw, Poland; konrad.futera@gmail.com (K.F.); askalski@mchtr.pw.edu.pl (A.S.); dan.janczak@gmail.com (D.J.); marcin.sloma@mchtr.pw.edu.pl (M.S.); maljakub@mchtr.pw.edu.pl (M.J.); 2 Institute of Microelectronics and Optoelectronics, Warsaw University of Technology, Koszykowa 75, 00-662 Warsaw, Poland; K.Kielbasinski@elka.pw.edu.pl; 3 Tele and Radio Research Institute, Ratuszowa 11, 03-450 Warsaw, Poland; aneta.arazna@itr.org.pl

**Keywords:** graphene nanoplatelets, graphene inks, inkjet printing, inks viscosity, dispersant agents

## Abstract

Inkjet printing is an excellent printing technique and an attractive alternative to conventional technologies for the production of flexible, low-cost microelectronic devices. Among many parameters that have a significant impact on the correctness of the printing process, the most important is ink viscosity. During the printing process, the ink is influenced by different strains and forces, which significantly change the printing results. The authors present a model and calculations referring to the shear rate of ink in an inkjet printer nozzle. Supporting experiments were conducted, proving the model assumptions for two different ink formulations: initial ink and with the addition of a dispersing agent. The most important findings are summarized by the process window regime of parameters, which is much broader for the inks with a dispersing agent. Such inks exhibit preferable viscosity, better print-ability, and higher path quality with lower resistivity. Presented results allow stating that proper, stable graphene inks adjusted for inkjet technique rheology must contain modifiers such as dispersing agents to be effectively printed.

## 1. Introduction

Inkjet printing is one of the most promising technologies and an attractive alternative to conventional technologies for the production of electronic devices [1,2,3]. Due to the large numbers of advantages such as possibility of direct and noncontact printing on various substrates, the simplicity of use in mass production and its low cost [1,2], it shows a wide range of potential applications, not only in electronics but also in medicine [4,5] or the textile industry [6,7].

The most important component of the inkjet ink, affecting of its physical properties, is a functional phase. In the production of conductive inks metal nanoparticles and carbon-based materials such as carbon nanotubes or graphene are commonly used. Graphene is a carbon nanoform which enjoys in recent times an enormous popularity. This material exhibits unique, remarkable electronic and mechanical properties and can be used in a variety of applications [1,8]. Accordingly, research related to the heterophasic inks based on various forms of graphene is widely described in the literature. The vast majority of research focuses on the more easily dispersible, but nonconductive material—graphene oxide (GO) and its chemical derivatives, such as reduced graphene oxide (rGO) [1,9,10,11,12,13,14]. However, presumably through substantial difficulties in its dispersion in solvents, properties of graphene nanoflakes (GNP) have not yet been thoroughly tested for inkjet inks. Only individual researchers describe heterophasic inks with GNP [15,16,17,18], often resulting in poor quality and high resistivity of printing paths and strips.

The preparation of a stable, homogeneous, conductive graphene ink is a huge challenge. Among many parameters (diameter and temperature of the nozzle, pulse amplitude and length and properties of the substrate), which influence repeatable and reliable inkjet printing process with high-quality patterns, the most important are the physical properties of the ink such as viscosity [1]. Ink viscosity strongly affects the form and volume of the ink droplet. Too high a value can result in clogging of the printer nozzle, and too low a value can cause in generation of an aerosol instead of a droplet [3,19].

Dispersing agents are the components influencing dispersion of particles in a vehicle, reducing their aggregation and sedimentation. The addition of these materials also affects significantly the rheology of the ink by reducing its viscosity and surface tension [20,21,22]. In the literature, we can find research results regarding application of the surfactants and dispersing agents in various inkjet inks, among others with: silver [21,23], cobalt [20], and nickel [24] nanoparticles as well as with carbon-nanotubes [25], GO or rGO [10,11]. There are also reports of attempts to disperse graphene flakes using surfactants or polymers. Wajid et al. [26] dispersed GNP in Poly (vinyl pyrrolidone). However, this author did not investigate whether the suspension, based on a viscous polymer, can be used as inkjet ink. There is no research results related precisely to application of dispersing agents and their influences on GNP inkjet inks viscosity and printability.

Moreover, although inkjet printing is a technology used for decades [27], there is a lack of the description of the physical effects influencing the ink in the nozzle during the printing process in the literature. It is possible to find a model of a capillary break-up of heterophase suspensions, explaining the thinning behavior of the inks [28]. However, when analyzing the viscosity of inks, it is very important to know also the value of shear rate acting on the ink droplet.

Derby at. al stated that “strain rates are expected to be in the range 10^3^–10^4^″ [29]. In other sources, we can find that the shear rates observed during inkjet printing are usually in the order of 10^5^ [2,30,31]. Therefore, it can be suspected that shear rates in an inkjet printer’s nozzle are very high. However, there is no calculation of the real value.

Therefore, it is desirable to study the effect of dispersing agents on the viscosity, stability, and printability of GNP inks, complemented by the model and calculations referring to the shear rate of ink in a printer nozzle. In this paper, we propose such approach along with the method to formulate conductive GNP inks for the inkjet printing.

## 2. Materials and Methods

### 2.1. Ink Formulation

**Figure 1:**
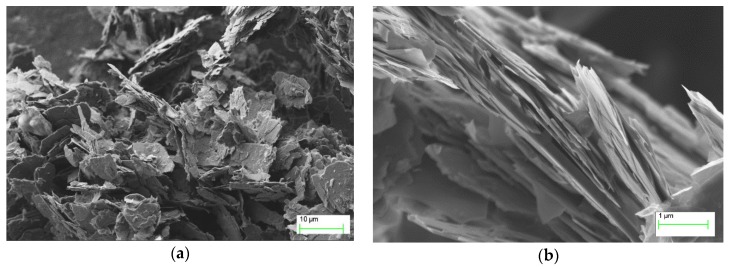
Scanning electron microscope pictures of graphene nanoplatelets used in the inks.

Materials for the experiments were prepared from three main components: conductive active phase (GNPs), vehicle (organic solvent) and dispersing agents. Graphene nanoplatelets, presented in Figure 1, were commercially acquired from Institute of Electronic Materials Technology, Warsaw, Poland. Their average thickness was 10 nm with the average diameter of 8 μm.

The inks were formulated using a base mixture of glycol and ethyl alcohol (50:50 wt % ratio). GNPs were added (0.5 wt %) to the base solution and sonicated for 2 h using the ultrasonic bath from InterSonic Company, model IS-1K, Olsztyn, Poland. The aim was to break agglomerates and obtain a uniform dispersion of nanoparticles. To increase the effect, before the sonication of the second ink, we have mixed GNPs with a very small amount (1 wt %) of commercial polymer dispersing agent AKM-0531 from NOF Corporation, Tokyo, Japan. It is one of functional polymers that have polyoxyalkylene groups on side chain and ionic groups on main chain dispersant both for inorganic and organic powders.

### 2.2. Rheology Measurements and Printing Process

Viscosity measurements of prepared inks were measured on BROOKFIELD DV2T cone-plate viscometer (Middleboro, MA, USA), with CP-40 cone and were conducted in a temperature range from 15 to 45 °C, controlled using the Polyscience Ultrathermostat. The Rheocalc T program measured the viscosity at different totation speeds: from 0.5 to 60 rpm. Each point was measured within 5 s.

Printing process was performed with use of custom piezoelectric inkjet printer with MD-K-130 printhead from Micro Drop (Norderstedt, Germany) with single 50 µm nozzle. The pulse length was tested in the range 30–360 µs. The graphene inks were printed on 100 µm thick Polyethylene terephthalate (PET) foil from DuPont. During the printing process, this substrate was placed on a table heated to 50 °C.

## 3. Results and Discussion

### 3.1. Calculation of the Shear Rate Acting on the Ink Inside the Nozzle

**Figure 2:**
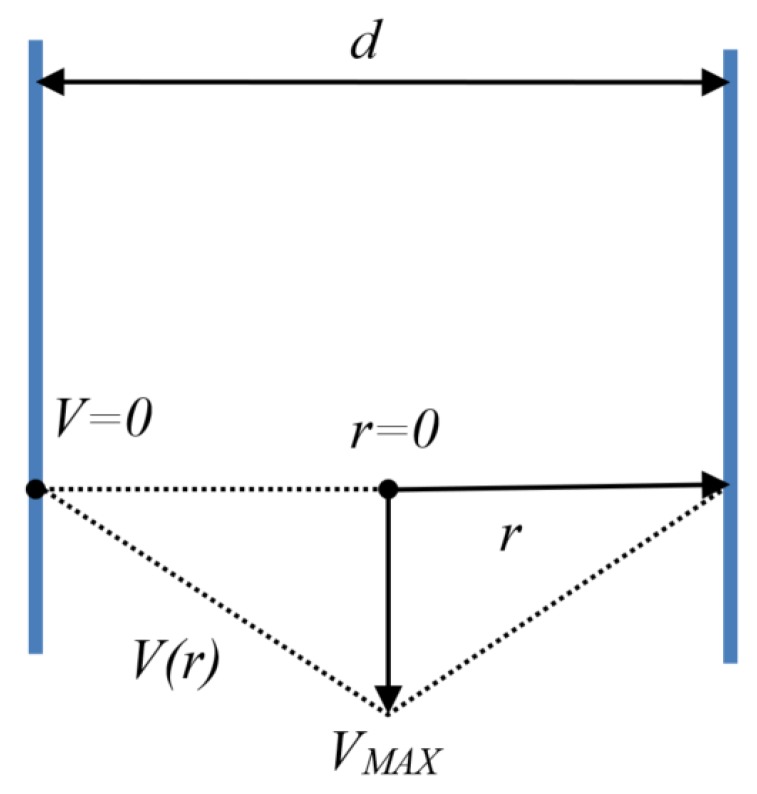
Model of ink velocity distribution inside the inkjet nozzle.

Our calculations, based on the assumptions outlined below, were made to estimate the minimum value of shear rate for ejected fluid inside the nozzle. This is of great importance when analyzing viscosity values on the viscosity curve. The velocity distribution inside the nozzle has a triangle shape approximation with zero at the nozzle’s walls, rising linearly and reaching the maximum at the orifice, as shown in Figure 2. Triangular speed profile allows to state single mean shear rate for the whole fluid volume, that could not be done using sophisticated parabolic speed profile.

Assuming that the inkjet nozzle is a cylindrical shape, the cross-sectional area is denoted by the Equation (1) and the perimeter for given radius *r*—the Equation (2). While using the formula for shear rate according to the Couette flow (3) the velocity of ink on a given radius can be calculated. Therefore, a value of the mean velocity of the fluid at the cross-sectional area of a cylindrical nozzle can be calculated (4). Moreover, due to energy loss during segmentation of a fluid stream into a droplet, the velocity of the ejected droplet *V_DROP_* cannot be higher than the mean velocity of the fluid inside the nozzle *V_MEAN_* (5).

(1) $$S=\pi \;\times \;\left(\frac{d^{2}}{4}\right)$$

(2) δS(r)=2πr

(3) $$V\left(r\right)=\gamma \;\times \;\left|\frac{d}{2}-r\right|$$

(4) $$V_{MEAN}=\frac{\int_{r=0}^{\frac{d}{2}}\delta S\left(r\right)\;\times \;V\left(r\right)}{S}dr=0.166\;\times \;\gamma \;\times \;d$$

(5) $$V_{DROP}\leq V_{MEAN}$$

(6) $$\;\gamma \geq \frac{V_{DROP}}{0.166\;\times \;d}$$

(7) $$\gamma \geq 6\;\times \;10^{5}\frac{1}{s}$$

where: *V_MAX_—*maximum velocity at the center of the nozzle, *d—*nozzle diameter*, S—*nozzle cross-sectional area, *δS(r)—*perimeter for given radius *r*, *V(r)—*velocity of the point for given radius, *V_MEAN_—*mean velocity of the fluid at cross-sectional area, *V_DROP_—*droplet velocity, *γ—*shear rate.

Value of the *V_DROP_* should not be lower than 5 m/s [32,33] and the diameter of the nozzle used in this research is *d* = 50 µm, what gives us calculated shear rate *γ* greater than or equal $6\;\times \;10^{5}\frac{1}{s}$, according to the Equation (6). This value is consistent with the publications [2,30,31] reporting that shear rates during inkjet printing should be close to the order of $10^{5}\frac{1}{s}$. This very high value often exceeds the measurement capabilities of a regular viscometer. However, in case of shear thinning fluids, viscosity value can be read for the last measured point, for shear rates in which the viscosity value has already stabilized.

### 3.2. The Viscosity of Inks

**Figure 3:**
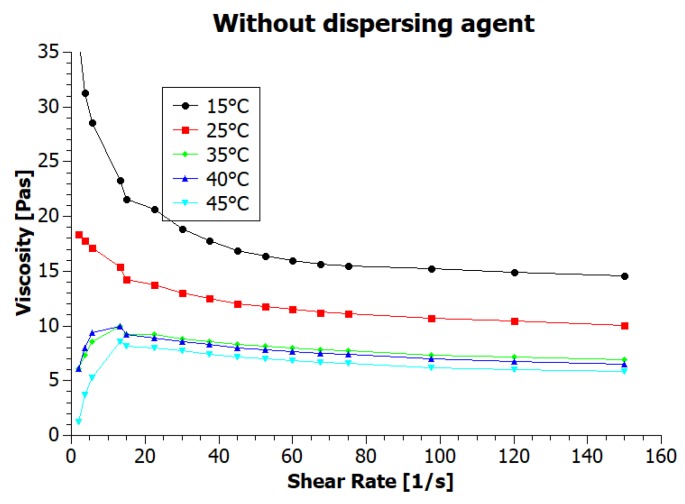
Viscosity curves of graphene ink without dispersing agent at various temperatures.

**Figure 4:**
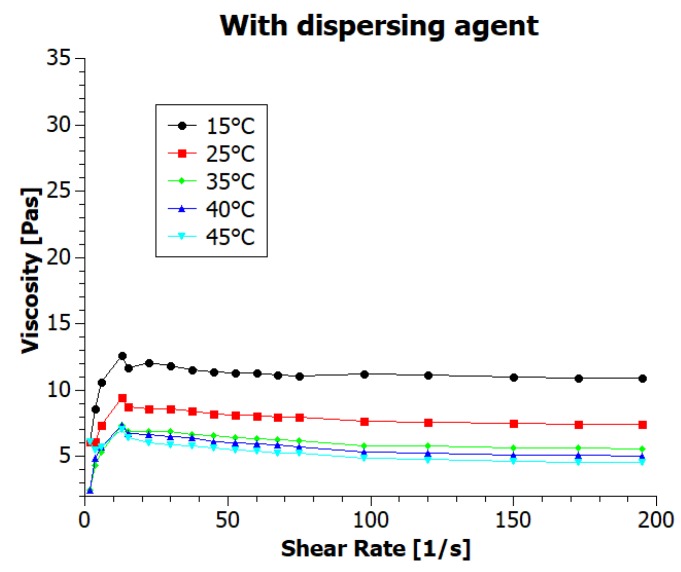
Viscosity curves of graphene ink with dispersing agent at various temperatures.

Numerous parameters affect the viscosity, which is one of the critical properties of printing inks [2] and the temperature is here of great importance*—*viscosity value decreases when the temperature increases. Often heated ink container prevents from clogging the nozzle during printing of higher viscosity inks, lowering their viscosity. To test the temperature dependency of the ink viscosity we have conducted rheological measurements in various temperatures, from 15 to 45 °C, with results presented in Figure 3 and Figure 4 (inks viscosity curves*—*viscosities values as a function of shear rate).

For both inks, the viscosity decreased with increasing shear rate, showing typical pseudoplastic (shear-thinning) fluid behavior. It is characterized by decrease in the viscosity for shear rate within a range of 0–100 s^−1^ [2]. For higher shear rates value, viscosity is stable. According to assumptions, temperature increase also resulted in a decrease of the viscosity. The ink viscosity values at 25 °C were approximately 10 and 7.5 mPas. Shi et al. [9] state that “ideal ink for inkjet printing is required to have a viscosity between 1 and 20 mPas” and Li et al. [15] specify that inkjet printing typically requires an ink viscosity of approximately 10 mPas. Therefore, we can say that the viscosity of our inks meets the requirements of inkjet printing technique. The viscosity values in a wide range of temperatures are presented in Table 1.

The viscosity of the ink containing the dispersing agent (GNP2) for all temperatures was lower than for the ink without dispersing agent (GNP1). This results are caused by better dispersion of graphene nanoparticles thanks to the addition of a dispersing agent and resulted in better printability. Changes in viscosity corresponding to changes in temperature for both inks were comparable.

One of the models describing the viscosity curves of generalized Newtonian liquids in shear flow is the Carreau Model (8) [1]. It describes the behavior of fluids exhibiting shear thinning behavior. In the low shear range the liquid acts as a Newtonian fluid, while at high-thinning properties are determined by the Power Law rule, with the coefficient of dilution expressed in the viscosity loss multiplicities per shear rate increase [1,2,3].

(8) $$\mu \left(\gamma \right)=\mu_{\infty }+(\mu_{0}-\mu_{\infty }){\left(1+{\left(\chi \gamma \right)}^{2}\right)}^{\frac{n-1}{2}}$$

where: *µ*_∞_*—*viscosity at infinite shear rate, *µ*_0_*—*viscosity at zero shear rate, *χ—*relaxation time, *n—*power index

**Figure 5:**
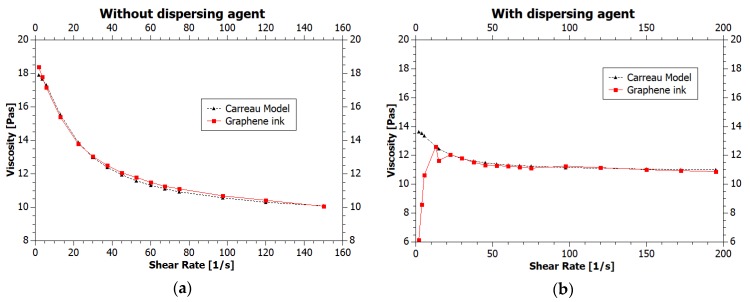
Graphene viscosity curves with fitted Carreau Model curve (**a**) graphene ink without dispersing agent; (**b**) graphene ink with dispersing agent.

The Carreau Model was fitted to the viscosity curves obtained at 25 °C temperature. The results are presented in Figure 5.

Matching the model to the viscosity curve of the ink without a dispersing agent confirms the correct adaptation of the used method and allows to characterize the ink as shear thinning liquid. In second case, the fit of the curve characterizing the viscosity of the ink with the dispersing agent is less accurate, especially at low shear rates. However, the model is well-suited in the area of high stresses, occurring in droplet formation regime.

### 3.3. Calculation of the Temperature Dependence of Inks Viscosity Parameters

A thermodynamic model and research on the dependence of enthalpy and viscosity of liquid mixtures are fundamental for the understanding of different types of intermolecular interactions in these mixtures [34]. Temperature dependence of inkjet inks viscosity can be described by the Arrhenius model equation:

(9) $$\eta =\eta_{0}\;\times \;\exp\left(\frac{E}{RT}\right)$$

where: *η—*ink viscosity, *η*_0_*—*coefficient characteristic for the liquid, *E—*activation energy, *R—*universal gas constant, *T—*temperature.

**Figure 6:**
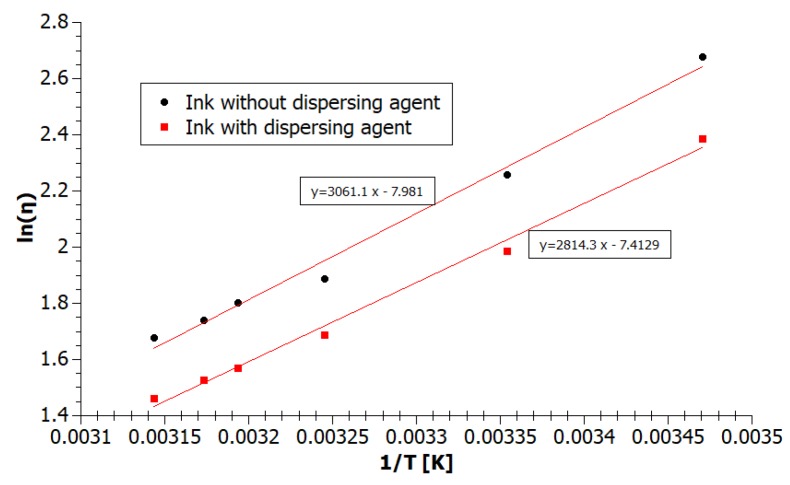
Diagram of viscosity as a linear function of the reciprocal of temperature.

We have determined the two parameters of the temperature dependence of viscosity: activation energy *E* and special coefficient characteristic for the liquid *η_0_* using transformed Equation (9). The new Formula (10) and following graph, presented in Figure 6, show the viscosity as a linear Function (11) of the reciprocal of temperature.

(10) $$\;ln\eta =\frac{E}{R}\;\times \;\frac{1}{T}+ln\eta_{0}\;\;$$

(11)  y=A × x+B  

(12) $$\;A=\frac{E}{R},\;\;\;B=ln\eta_{0}\;$$

Using the least squares method for the inks viscosity values *η* at different temperatures *T* we have calculated the coefficients of the functions *A* and *B*, and next temperature parameters *E* and *η*_0_ (12).

Activation energy *E* thus expresses the molar energy required to overcome the intermolecular forces that inhibit the shifting of the layers at the flow of liquid*—*the activation energy of the viscous flow [35], which can be also related to the enthalpy of vaporization at the same pressure [34]. According to the assumption, the ink containing the dispersing agent (GNP2) has a lower activation energy*—*23.4 kJ/mol than the ink without a dispersing agent (GNP1)*—*25.5 kJ/mol. Consequently, we can state, that the cohesion of the ink GNP2 and the frequency of reactions between the ink molecules is lower than for ink GNP1. The ink with the lower value of activation energy should allow easier drops formation, without problems with detachment of the drops and satellites creating.

### 3.4. Influence of Pulse Length on Drop Formation

Printability of our inks was estimated basis on the width of process window*—*the range of pulse parameters, in which the correct droplet could be formulated.

**Figure 7:**
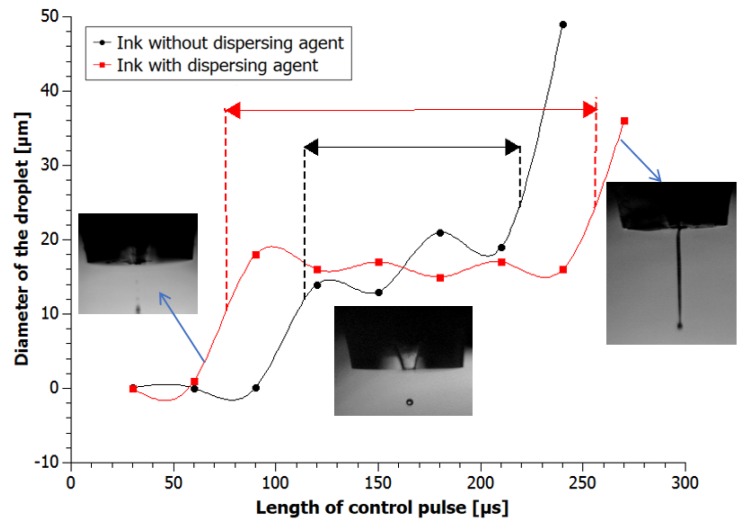
Diameter of inks droplets depending on the length of the pulse along with the selected technological window.

Inks droplet diameters were measured for the control pulse length from 30 to 360 µs. From the Figure 7, we see that in the range 30–150 µs GNP2 droplets were larger than droplets of GNP1 and, on the contrary, under 150 µs they were smaller. In the central region of pulse range droplet diameters were 15–18 μm for GNP2 and 13–21 μm for GNP1. However, the single droplet with a repetitive, stable diameter was observed only in the area restricted by the optimal parameter region (process window) marked in Figure 6. A lower value of the pulse length resulted in the lack of the possibility of forming a drop or with aerosol instead of a drop. Higher value resulted in a constant flow of the ink. The window of recommended pulse length for the inks with dispersing agent was much wider than for the inks without dispersing agent. For the GNP1 ink, it was 115–220 µs and for GNP2*—*75–255 µs.

As observed, the ink containing a viscosity modifier allows for wider range of printing parameters.

### 3.5. Printing Paths

**Figure 8:**
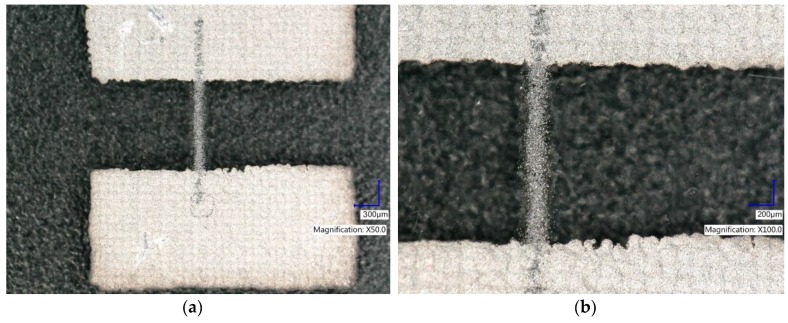

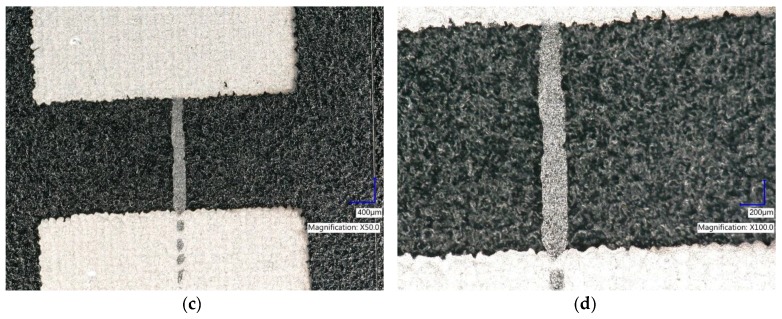
Graphene printed paths (**a**,**b**) produced using nondispersant ink (**c**,**d**) produced using ink with dispersing agent. The graphene paths were printed using piezoelectric inkjet printer with parameters: nozzle diameter: 50 µm, voltage: 40–50 V, pulse length: 150–200 µs.

We can clearly observe the differences between the quality of patterns obtained using the non-dispersant ink (Figure 8a,b) and the ink with dispersing agent, on the favor of the modified ink (Figure 8c,d).

Patterns printed with graphene nondispersant ink have blurry edges. At the edges, instead of a uniform layer, single agglomerates of graphene flakes are visible. The resulting sample is non-homogeneous and exhibiting higher sheet resistivity of 125 $\frac{k\Omega }{□}$. Due to better dispersion of GNPs and good rheology of second ink, no problems were observed with droplet formation during its ejection from the nozzle. The ink was properly distributed on the substrate and form a good quality pattern. This also led to an increase in the conductivity of the layer, reducing the resistance to 90 $\frac{k\Omega }{□}$. This is a very low value, in comparison to the results received by other researchers for GNP inkjet paths. Torrisi et al. [16] obtained paths with resistance about 500 $\frac{k\Omega }{□}$ on pristine glass. Obtained by us results of sheet resistivity are also more than two times better than received by Li et al. (15), that printed paths on pristine glass and Kapton with sheet resistivity about 200 $\frac{k\Omega }{□}$.

## 4. Conclusions

In summary, we have investigated the relationship between inkjet inks printability and rheological properties. It was found that the calculated minimal shear rate for the ink inside the printing nozzle during ejection has a very high value of more than 6 × 10^5^$\frac{1}{s}$ . Two different graphene inks dedicate to inkjet printing technology were tested: base and modified with the dispersing agent. According to the presented calculations of the temperature influence on the viscosity, for both tested graphene inks viscosity decreased with increasing temperature with the same trend for both inks. Moreover, the viscosity value of the ink with dispersing agent is lower than for the ink without them. Viscosity value of the formulating inks was about 10 mPas, simultaneously meeting the requirements of inkjet printing technique. The process window for the ink with dispersing agent was much wider for ink than without them, which means the ink was more effective to print. The cause of this effect was not only lower viscosity but also the distinctly better dispersion of graphene nanoparticles in the ink. Moreover, paths produced using this ink has better quality and conductivity; they attain a sheet resistivity around 90 $\frac{k\Omega }{□}$.

The graphene ink described in the article has already been used, among others, in the production of full inkjet printed microwave module. The obtained resistive divider was printed on a flexible, transparent Kapton foil, using graphene heterophase inks [31].

The results indicate that rheological behavior and dispersion state of graphene inks used in inkjet printing significantly affects graphene inks printability and the quality of a produced pattern, and so it should be carefully controlled. Therefore, it can be stated that the surfactant is an absolutely necessary element of graphene inks to ensure stable suspension with adjusted for inkjet technique rheology and conductive paths.

## Figures and Tables

**Table 1:** The viscosity values of the graphene inks.

Temperature (°C)	GNP1 Ink Viscosity (mPas)	GNP2 Ink Viscosity (mPas)
15	14.56	11.00
25	10.07	7.48
35	6.54	5.06
45	5.82	4.46

## References

[1] Huang L., Huang Y., Liang J., Wan X., Chen Y. (2011). Graphene-based conducting inks for direct inkjet printing of flexible conductive patterns and their applications in electric circuits and chemical sensors. Nano Res..

[2] Woo K., Jang D., Kim Y., Moon J. (2013). Relationship between printability and rheological behavior of inkjet conductive inks. Ceram. Int..

[3] Zhang W.L., Choi H.J., Ko H.S., Kwon K.S. (2015). Inkjetting and rheological behavior of a silica particle suspension. J. Ind. Eng. Chem..

[4] Scoutaris N., Alexander M.R., Gellert P.R., Roberts C.J. (2011). Inkjet printing as a novel medicine formulation technique. J. Control. Release.

[5] Tricomi B.J., Dias A.D., Corr D.T. (2016). Stem cell bioprinting for applications in regenerative medicine. Ann. N. Y. Acad. Sci..

[6] Wang C., Wang L., Huang Y., Meng Y., Sun G., Fan Q., Shao J. (2017). Fabrication of reactive pigment composite particles for blue-light curable inkjet printing of textiles. RSC Adv..

[7] Stempien Z., Rybicki E., Rybicki T., Lesnikowski J. (2015). Inkjet-printing deposition of silver electro-conductive layers on textile substrates at low sintering temperature by using an aqueous silver ions-containing ink for textronic applications. Sens. Actuators B Chem..

[8] Mao H.Y., Laurent S., Chen W., Akhavan O., Imani M., Ashkarran A.A., Mahmoudi M. (2013). Graphene: Promises, Facts, Opportunities, and Challenges in Nanomedicine. Chem. Rev..

[9] Shin K.Y., Hong J.Y., Jang J. (2011). Micropatterning of graphene sheets by inkjet printing and its wideband dipole-antenna application. Adv. Mater..

[10] Lee C.L., Chen C.H., Chen C.W. (2013). Graphene nanosheets as ink particles for inkjet printing on flexible board. Chem. Eng. J..

[11] Wang J., Fang Z., Zhu H., Gao B., Garner S., Cimo P., Barcikowski Z., Mignerey A., Hu L. (2014). Flexible, transparent, and conductive defrosting glass. Thin Solid Films.

[12] Le L.T., Ervin M.H., Qiu H., Fuchs B.E., Lee W.Y. (2011). Graphene supercapacitor electrodes fabricated by inkjet printing and thermal reduction of graphene oxide. Electrochem. Commun..

[13] Dua V., Surwade S.P., Ammu S., Agnihotra S.R., Jain S., Roberts K.E., Park S., Ruoff R.S., Manohar S.K. (2010). All-organic vapor sensor using inkjet-printed reduced graphene oxide. Angew. Chem. Int. Ed..

[14] Zhang L., Liu H., Zhao Y., Sun X., Wen Y., Guo Y., Gao X., Di C.A., Yu G., Liu Y. (2012). Inkjet printing high-resolution, large-area graphene patterns by coffee-ring lithography. Adv. Mater..

[15] Li J., Ye F., Vaziri S., Muhammed M., Lemme M.C., Östling M. (2013). Efficient inkjet printing of graphene. Adv. Mater..

[16] Torrisi F., Torrisi T., Hasan W., Wu Z., Sun A., Lombardo T., Kulmala G.-W., Hsieh S., Jung F., Bonaccorso P. (2012). Inkjet-Printed Graphene Electronics. ACS Nano.

[17] Secor E.B., Gao T.Z., Islam A.E., Rao R., Wallace S.G., Zhu J., Putz K.W., Maruyama B., Hersam M.C. (2017). Enhanced Conductivity, Adhesion, and Environmental Stability of Printed Graphene Inks with Nitrocellulose. Chem. Mater..

[18] Secor E.B., Prabhumirashi P.L., Puntambekar K., Geier M.L., Hersam M.C. (2013). Inkjet printing of high conductivity, flexible graphene patterns. J. Phys. Chem. Lett..

[19] Caglar U. (2009). Studies of Inkjet Printing Technology with Focus on Electronic Materials.

[20] Nelo M., Sowpati A., Palukuru V.K., Juuti J., Jantunen H. (2010). Formulation of Screen Printable Cobalt Nanoparticle Ink for High Frequency Applications. Prog. Electromagn. Res..

[21] Kamyshny A. (2011). Metal-based Inkjet Inks for Printed Electronics. Open Appl. Phys. J..

[22] Gamota D., Brazis P., Kalyanasundaram K., Zhang J. (2004). Printed Organic and Molecular Electronics.

[23] Chen C.N., Huang C.T., Tseng W.J., Wei M.H. (2010). Dispersion and rheology of surfactant-mediated silver nanoparticle suspensions. Appl. Surf. Sci..

[24] Tseng W.J., Chen C.N. (2006). Dispersion and rheology of nickel nanoparticle inks. J. Mater. Sci..

[25] Moore V.C., Strano M.S., Haroz E.H., Hauge R.H., Smalley R.E., Schmidt J., Talmon Y. (2003). Individually wuspended wingle-walled carbon nanotubes in various surfactants. Nano Lett..

[26] Wajid A.S., Das S., Irin F., Ahmed H.S.T., Shelburne J.L., Parviz D., Fullerton R.J., Jankowski A.F., Hedden R.C., Green M.J. (2012). Polymer-stabilized graphene dispersions at high concentrations in organic solvents for composite production. Carbon N. Y..

[27] Heinzl J., Hertz C.H. (1985). Inkjet Printing. Adv. Eletron. Electron Phys..

[28] McIlroy C., Harlen O.G. (2014). Modelling capillary break-up of particulate suspensions. Phys. Fluids.

[29] Derby B., Reis N. (2003). Inkjet Printing of Highly Loaded Particulate Suspensions. MRS Bull..

[30] Tuladhar T.R., Mackley M.R. (2008). Filament stretching rheometry and break-up behaviour of low viscosity polymer solutions and inkjet fluids. J. Nonnewton. Fluid Mech..

[31] De Gans B.-J., Duineveld P.C., Schubert U.S. (2004). Inkjet printing of polymers: State of the art and future developments. Adv. Mater..

[32] Dong H., Carr W.W., Morris J.F. (2006). An experimental study of drop-on-demand drop formation. Phys. Fluids.

[33] Castrejón-Pita J.R., Martin G.D., Hoath S.D., Hutchings I.M. (2008). A simple large-scale droplet generator for studies of inkjet printing. Rev. Sci. Instrum..

[34] Messaâdi A., Dhouibi N., Hamda H., Belgacem F.B.M., Adbelkader Y.H., Ouerfelli N., Hamzaoui A.H. (2015). A New Equation Relating the Viscosity Arrhenius Temperature and the Activation Energy for Some Newtonian Classical Solvents. J. Chem..

[35] Dyre J.C., Olsen N.B., Christensen T. (1996). Local elastic expansion model for viscous-flow activation energies of glass-forming molecular liquids. Phys. Rev. B.

